# Inhibition of Mast Cell Degranulation in Atopic Dermatitis by Celastrol through Suppressing MRGPRX2

**DOI:** 10.1155/2023/9049256

**Published:** 2023-01-18

**Authors:** Ciyu Yao, Wenzhen Ye, Mengxue Chen

**Affiliations:** Department of Dermatology, Fuzhou Hospital of Traditional Chinese Medicine Affiliated to Fujian University of Traditional Chinese Medicine, No. 102, Gudong Road, Fuzhou, 350000 Fujian Province, China

## Abstract

**Background:**

Atopic dermatitis is a common dermatological disease, and mast cell degranulation is believed to be related with the progression of atopic dermatitis. Mas-related G protein-coupled receptor-X2 (MRGPRX2), and calcium release-activated calcium channel protein 1-2 (ORAI-1, ORAI-2) are involved in mast cell degranulation. Celastrol is an active monomer of Tripterygium wilfordii, and it presents an antiatopic role.

**Methods:**

2,4-Dinitrofluorobenzene (DNFB) and compound 48/80 (C 48/80) were used to establish a slow and acute scratching animal model, respectively. Hematoxylin-eosin and toluidine blue staining was used to investigate tissue injury. Inflammatory factor concentration was measured with ELISA. The expression of MRGPRX2, ORAI-1, and ORAI-2 was detected with immunohistochemistry (IHC) staining. Gene expression profiling and microRNA array were performed to investigate gene differential expression.

**Results:**

Celastrol greatly inhibited atopic dermatitis-related tissues injury, mast cell production, histamine release, scratching level, inflammatory factor expression, and activation of MRGPRX2/ORAI axis in the DNFB-induced atopic dermatitis model. The influence of Celastrol on atopic dermatitis was remarkably reversed by overexpression of MRGPRX2.

**Conclusion:**

We found that the improvements of atopic dermatitis caused by Celastrol were reversed by treatment with MRGPRX2^OE^, indicating that Celastrol might affect atopic dermatitis through MRGPRX2. This study might provide a novel thought for the prevention and treatment of atopic dermatitis by regulating MRGPRX2.

## 1. Introduction

Atopic dermatitis is a common dermatological disease. The repeated inflammation, stubborn itching, and skin barrier damage caused by atopic dermatitis can seriously affect the quality of life of patients [1, 2]. The pathogenesis of atopic dermatitis is not very clear. It may be caused by autoimmunity, genetic factors, environmental factors, skin barrier dysfunction, bacterial infection, and other pathogenic factors [3, 4]. At present, the treatment of atopic dermatitis is mainly through local medication or oral administration to reduce inflammation and itching [5]. Topical glucocorticoid is a common local treatment for atopic dermatitis, but it may cause adverse reactions such as skin atrophy, telangiectasia, pigmentation, and systemic absorption, which limits its application [6].

Mast cells activated in atopic dermatitis release a large number of inflammatory cytokines, histamine, and protease through degranulation [7, 8] and play an important role in regulating inflammatory response and itching [9, 10]. Mas-related G protein-coupled receptor-X2 (MRGPRX2) is specifically expressed by skin mast cells and is considered a new marker for regulating mast cell degranulation, chronic inflammation, and anaphylaxis [11]. MRGPRX2 may utilize calcium release-activated calcium channel protein 1 (ORAI-1) and calcium release-activated calcium channel protein 2 (ORAI-2) to promote mast cell degranulation [12]. The regulation of calcium influx by ORAI-1 is related to RhoA/Rock/MAPK signaling, which is closely related to mast cell degranulation [13]. However, the mechanism by which ORAI-1 and ORAI-2 interact with MRGPRX2 to regulate mast cell degranulation is unclear.

Celastrol, as an active monomer of Tripterygium wilfordii, can play an antiatopic role by reducing calcium ion influx, *β*-hexosaminidase, histamine, Th2 cytokines, and cell adhesion molecules [14, 15]. However, whether Celastrol regulates atopic dermatitis through MRGPRX2 has not been reported. The purpose of this study is to explore whether the molecular mechanism of Celastrol intervention on mast cell degranulation in atopic dermatitis is related to the interaction of MRGPRX2/ORAI.

In the present study, a 2,4-dinitrofluorobenzene- (DNFB-) induced atopic dermatitis animal model was establish, and compound 48/80 (C 48/80) was used to establish an acute scratching animal model. We demonstrated that Celastrol could inhibit atopic dermatitis through suppressing MRGPRX2/ORAI axis. This research might provide a new insight for the prevention and treatment of atopic dermatitis.

## 2. Methods

### 2.1. Establishment of a Slow Atopic Dermatitis Animal Model with DNFB

All the experiments were approved by the Ethical Committee of Fujian University of Traditional Chinese Medicine. BALB/c mice (18-25 g, Charles River, Beijing, China) were used in this study. Totally, 4 groups were set in this research with 10 mice in each group. The slow atopic dermatitis animal model was established with DNFB (#D1529, Sigma). 0.8% Na_2_S solution was applied to 2.5 cm × 2.5 cm back skin of mice for hair preparation, and 100 *μ*L of 0.5% DNFB solution was applied on the back of the mice the next day. 100 *μ*L of 0.5% DNFB solution was used to daub the back of the mice once a day for 14 days. The mice in the control group were treated with PBS with the same way. The animals in the DNFB+Celastrol and DNFB+Celastrol+MRGPRX2^OE^ group were treated with 0.5% DNFB solution as described above. Meanwhile, the mice in the DNFB+Celastrol group were treated with Celastrol (3 mg/kg, #C0869, Sigma) gavage once a day. The mice in the DNFB+Celastrol+MRGPRX2^OE^ group were treated with Celastrol (3 mg/kg) gavage once a day and injected with 50 *μ*L MRGPRX2^OE^ vectors (1 × 10^8^ transfection units/mL) through the tail vein once a day. At the 14^th^ day, the mice were sacrificed, and the skin tissues were collected for the following experiments. The scratch bouts were recorded at the 0, 3, 6, 9, and 12 days. Each recording time lasts for 30 min, and scratch bout times were recorded.

### 2.2. Establishment of an Acute Atopic Dermatitis Animal Model with C 48/80

The mice in the C 48/80 (#C2313, Sigma) and control group were treated with PBS (3 mg/kg) gavage once a day for 7 days. The mice in the C 48/80+Celastrol group were treated with Celastrol (3 mg/kg) gavage once a day for 7 days. The mice in the C 48/80+Celastrol+MRGPRX2^OE^ group were treated with Celastrol (3 mg/kg) gavage once a day and injected with 50 *μ*L MRGPRX2^OE^ vectors (1 × 10^8^ transfection units/mL) through the tail vein once a day for 7 days. Then, an acute atopic dermatitis animal model was established through intradermal injection of C 48/80 (50 *μ*g, 25 *μ*L). After the intradermal injection of C 48/80, scratch bout times were recorded immediately for 30 min. The animals in the control group were injected with the same amount of PBS.

### 2.3. Detection of Histamine

The serum level of histamine was measured with related commercial kit purchased from Nanjing Jiancheng Bioengineering Institute (#H171, Nanjing, China) according to instructions.

### 2.4. Immunohistochemistry (IHC) Staining

Paraffin sections were dewaxed firstly. Tissue sections were placed in EDTA antigen repair buffer and placed in a microwave oven for antigen repair (5 min). PBS (#ABA212278, Hyclone) was washed three times (5 min/time). The sections were put into 3% hydrogen peroxide solution and incubated for 25 min at room temperature in the dark to block endogenous peroxidase. The slides were washed three times in PBS (5 min/time). BSA was used and blocked for 30 min. The primary antibody was added to the sections, and the sections were incubated overnight at 4°C in a wet box. After washing three times in PBS, secondary antibody was added to incubate sections for 50 min at room temperature. Sections were incubated with DAPI dye for 5 min. Sections were observed under a fluorescence microscope, and images were collected. The antibodies used in this study were listed as follows: rabbit anti-MRGPRX2 antibody (ab237047, Abcam), mouse anti-Orai1 antibody (#SAB3500126, Sigma), and rabbit anti-Orai2 antibody (#HPA055137, Sigma).

### 2.5. Detection of TNF-𝑎, IL-6, IL-1*β*, and IL-31

The serum levels of TNF-𝑎 (#PT513), IL-6 (#PI326), and IL-1*β* (#PI301) were measured with related commercial kits purchased from Beyotime (Shanghai, China) according to instructions. The concentration of IL-31 (#ab243681) was detected with related commercial kit purchased from Abcam (UK).

### 2.6. Hematoxylin and Eosin (HE) Staining

Paraffin sections were dewaxed firstly, and the sections were successively put into xylene I for 20 min, xylene II for 20 min, absolute ethanol for 5 min, and 75% alcohol for 5 min. The sections were stained with hematoxylin staining solution (#G1004, Servicebio) for 3 min and washed with tap water. The sections were dehydrated with 85% and 95% gradient alcohol for 5 min and stained with eosin staining solution (#G1001, Servicebio) for 5 min. The sections were put into anhydrous ethanol for 5 min, xylene I for 5 min, and xylene II for 5 min. The neutral gum was sealed. The slides were observed with a microscope (Carl Zeiss, Germany).

### 2.7. Gene Expression Profiling

Total RNA from each sample was quantified using the NanoDrop ND-1000, and the RNA integrity was assessed using standard denaturing agarose gel electrophoresis. Agilent Array platform was employed. The sample preparation and microarray hybridization were performed based on the manufacturer's standard protocols. Briefly, total RNA from each sample was amplified and transcribed into fluorescent cRNA with the use of the manufacturer's Agilent's Quick Amp Labeling protocol. The labeled cRNAs were hybridized onto the Whole Mouse Genome Oligo Microarray (4 × 44K, Agilent Technologies). After washing the slides, the arrays were scanned by the Agilent Scanner G2505C. Agilent Feature Extraction software (version 11.0.1.1) was used to analyze acquired array images. Quantile normalization and subsequent data processing were performed using the GeneSpring GX v11.5.1 software package (Agilent Technologies). After quantile normalization of the raw data, genes were chosen for further data analysis. Differentially expressed genes were identified through fold change filtering.

### 2.8. MicroRNA Array

Total RNA was harvested using TRIzol (Invitrogen) and miRNeasy mini kit (QIAGEN) according to the manufacturer's instructions. After having passed RNA quantity measurement using the NanoDrop 1000, the samples were labeled using the miRCURY™ Hy3™/Hy5™ Power Labeling Kit and hybridized on the miRCURY™ LNA Array (v.18.0). Following the washing steps, the slides were scanned using the Axon GenePix 4000B microarray scanner. Scanned images were then imported into GenePix Pro 6.0 software (Axon) for grid alignment and data extraction. Replicated miRNAs were averaged, and miRNAs that haveintensities ≥ 30in all samples were chosen for calculating normalization factor. Expressed data were normalized using the median normalization. After normalization, differentially expressed miRNAs were identified through fold change filtering. Finally, hierarchical clustering was performed to show distinguishable miRNA expression profiling among samples.

### 2.9. Western Blot

The tissues were lysed firstly, and protein samples were separated with 10% SDS-PAGE. The proteins were transferred to nitrocellulose membrane (#Z358657, Millipore, USA). After blocking with 5% skim milk for 1 h, the related primary antibodies (1 : 1000) were used to culture membrane. Then, the membranes were incubated with secondary antibody (1 : 3000) for 2 h at room temperature. Finally, the protein grey was analyzed with ImageJ software. The antibodies used in this study were listed as follows: rabbit anti-MRGPRX2 antibody (ab237047, Abcam), mouse anti-Orai1 antibody (#SAB3500126, Sigma), and rabbit anti-Orai2 antibody (#ab180146, Sigma).

### 2.10. Statistical Analysis

Statistical difference was analyzed with at least 3 repeated experiments. The results were shown as the mean ± standard deviation. Data among different groups were analyzed with ANOVA. *p* < 0.05was considered to be statistically different.

## 3. Results

### 3.1. Inhibition of DNFB-Induced Atopic Dermatitis by Celastrol Was Reversed by Overexpression of MRGPRX2

**Figure 1:**
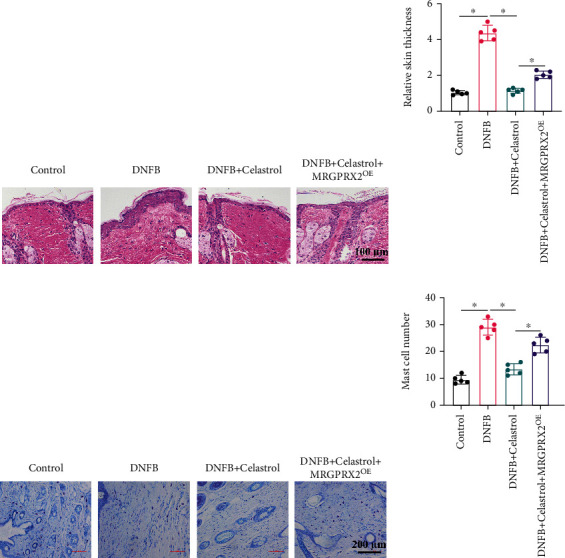
Inhibition of DNFB-induced atopic dermatitis by Celastrol was reversed by overexpression of MRGPRX2. (a) HE staining was performed to unfold the tissue damage. (b) The skin thickness was analyzed. (c) Toluidine blue staining was used to observe mast cells. (d) The number of mast cells was counted. Yellow arrows represent hair follicles, and red arrows represent mast cells. ^∗^ suggests *p* < 0.05.

To unfold the influence of Celastrol on atopic dermatitis, DNFB was used to induce an atopic dermatitis mice model. Compared with the control group, in the DNFB model group, the inflammatory cell infiltration (Figure 1(a)) and skin thickness (Figure 1(b)) in the model site were significantly increased. After Celastrol treatment, the infiltration of inflammatory cells was significantly alleviated, and skin thickness was decreased. However, overexpression of MRGPRX2 greatly inhibited the influence of Celastrol (Figures 1(a) and 1(b)). Meanwhile, after DNFB induction, mast cell number was significantly increased, but increased mast cells were suppressed by Celastrol (Figures 1(c) and 1(d)). In addition, the decreased mast cells by Celastrol were promoted by MRGPRX2^OE^ (Figures 1(c) and 1(d)).

### 3.2. Celastrol Greatly Inhibited Histamine and Scratch Bouts

**Figure 2:**
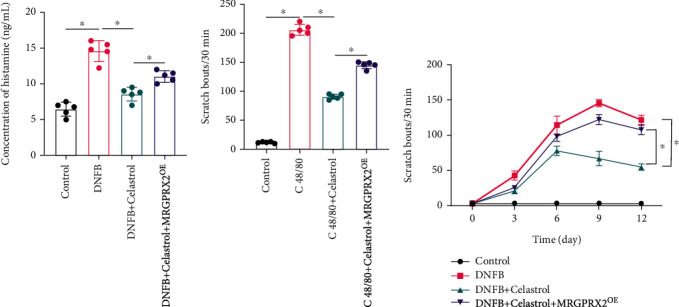
Celastrol greatly inhibited histamine and scratch bouts. (a) The level of histamine was analyzed. (b) An acute scratching animal model was established with C 48/80. (c) A slow scratching animal model was established with DNFB. ^∗^ suggests *p* < 0.05.

The level of histamine is closely related with itching degree. We found that histamine release was markedly promoted in the DNFB animal model (Figure 2(a)) compared with the control or DNFB+Celastrol group. However, after treatment with MRGPRX2^OE^, the level of histamine was significantly increased compared with the DNFB+Celastrol group (Figure 2(a)). An acute scratching animal model and slow scratching animal model were established with C 48/80 (Figure 2(b)) and DNFB (Figure 2(c)), respectively. The scratching bouts were greatly increased after C 48/80 or DNFB induction compared with the control group. However, administration with Celastrol remarkably suppressed the scratching levels, but simultaneous treatment with MRGPRX2^OE^ increased the times of scratching (Figures 2(b) and 2(c)).

### 3.3. The Inflammatory Factor Levels Were Suppressed by Celastrol

**Figure 3:**
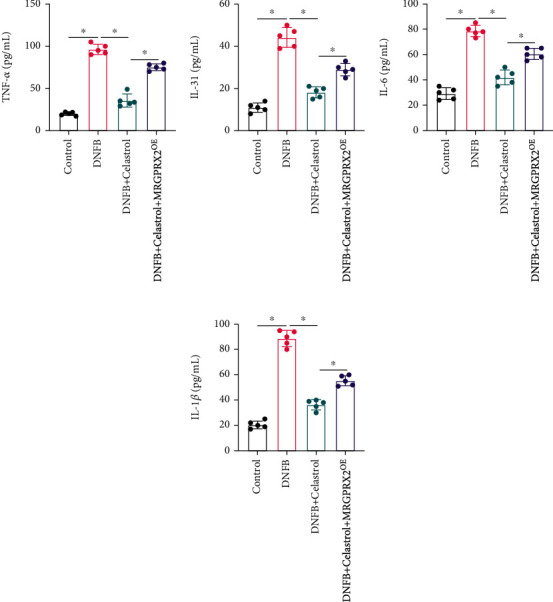
The inflammatory factor levels were suppressed by Celastrol. (a) The level of TNF-𝑎 was measured. (b) The level of IL-31 was measured. (c) The level of IL-6 was measured. (d) The level of IL-1*β* was measured. ^∗^ suggests *p* < 0.05.

The expression levels of inflammatory factors were measured. We found that the concentrations of TNF-𝑎 (Figure 3(a)), IL-31 (Figure 3(b)), IL-6 (Figure 3(c)), and IL-1*β* (Figure 3(d)) were markedly promoted in the DNFB group compared to the control or DNFB+Celastrol group. After treatment with Celastrol, the expression levels of inflammatory factors were greatly inhibited, and the influence of Celastrol on inflammatory factors was reversed by overexpression of MRGPRX2 (Figures 3(a)–3(d)).

### 3.4. The Activation of the MRGPRX2/ORAI Axis in the Atopic Dermatitis Animal Model Was Suppressed by Celastrol

**Figure 4:**
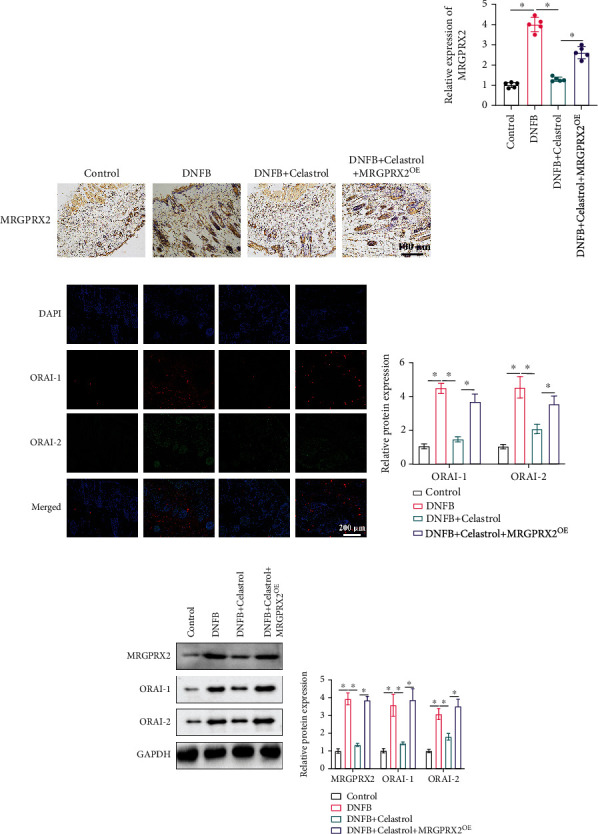
The activation of the MRGPRX2/ORAI axis in the atopic dermatitis animal model was suppressed by Celastrol. (a) The expression of MRGPRX2 in the skin tissue was measured with IHC staining. (b) The expression of MRGPRX2 was analyzed. (c) The levels of ORAI-1 and ORAI-2 in the skin tissue were detected with IHC staining. (d) The levels of ORAI-1 and ORAI-2 were analyzed. (e) The expression levels of the MRGPRX2/ORAI axis were measured with western blot; ^∗^ indicates *p* < 0.05.

The expression of the MRGPRX2/ORAI axis was evaluated by measuring the levels of MRGPRX2, ORAI-1, and ORAI-2 in skin tissue (Figures 4(a)–4(d)). After DNFB induction, the protein expression of MRGPRX2, ORAI-1, and ORAI-2 in skin tissue was remarkably promoted compared with the control group (Figures 4(a)–4(d)). However, the activation of the MRGPRX2/ORAI axis by DNFB was inhibited greatly by Celastrol. In addition, the suppression of the MRGPRX2/ORAI axis by Celastrol was greatly reversed by overexpression of MRGPRX2 (Figures 4(a)–4(d)). Similar influence of Celastrol and overexpression of MRGPRX2 on expression of the MRGPRX2/ORAI axis were observed and validated with western blot (Figure 4(e)).

### 3.5. Gene Differential Expression Analysis Was Performed

**Figure 5:**
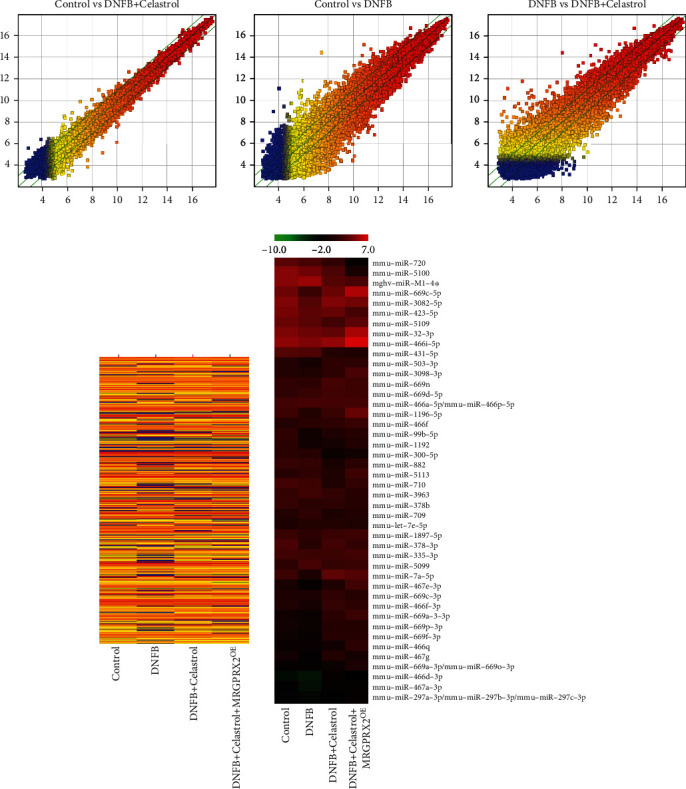
Gene differential expression analysis was performed. (a) Gene expression profiling was performed. (b) Gene expression heat map was analyzed. (c) MicroRNA array was performed.

Gene expression profiling and microRNA array were performed to investigate the gene differential expression. The default fold change value of the three green broken lines is 2 (Figure 5(a)). The point above and below the green line is the genefold change≧2.0 of the two comparative samples. If the expression difference between the two comparative samples is greater, the dispersion is greater. There was little difference in gene expression between the control and DNFB+Celastrol group (Figure 5(a)). However, significant gene expression differences were observed between the control, DNFB, and DNFB+Celastrol groups (Figure 5(a)). The gene expression heat map is presented in Figure 5(b). In addition, microRNA array was performed to investigate the expression differences of microRNAs in different groups (Figure 5(c)).

## 4. Discussion

Atopic dermatitis is a common chronic, recurrent, and itchy skin disease [2, 16]. Studies have shown that in atopic dermatitis, mast cells are recruited by chemokines and activated by IgE and non-IgE factors to produce a series of inflammatory factors [17]; interact with nerve cells, T cells, eosinophils, keratinocytes, etc.; and jointly participate in the process of skin itching and inflammation in atopic dermatitis [16, 18].

Skin pruritus is one of the main symptoms of atopic dermatitis, which reflects the severity of atopic dermatitis to a certain extent [19]. Mast cells are mononuclear immune cells that exist in skin and mucosal tissues [20]. They can be activated by allergens such as allogeneic proteins and chemical small molecule drugs to cause allergic reactions [21]. Its allergic mechanism is that the allergen binds to the immunoglobulin IgE receptor in mast cells, induces cells to release bioactive substances, produces degranulation, and causes physiological and pathological changes such as smooth muscle contraction, increased vascular permeability, and increased serous secretion [22]. In this study, we found that both acute scratching and slow scratching caused by C 48/80 and DNFB, respectively, could be greatly alleviated by Celastrol. However, the relief of scratching by Celastrol was significantly reversed by overexpression of MRGPRX2, indicating that Celastrol might regulate scratching through inhibiting MRGPRX2.

Mast cells can act as both effector cells and immune regulatory cells during the occurrence and development of atopic dermatitis [23, 24]. Mast cells regulate and recruit the chemotaxis of other inflammatory cells to further promote the process of atopic dermatitis [25, 26]. Histamine is a receptor that is released when mast cells activate sensory nerve endings and release neuropeptides. Mast cell activation leads to degranulation, vasodilation, plasma extravasation, and recruitment of immune cells [27, 28]. Mast cell activation is involved in the pathogenesis of pain and many chronic inflammatory diseases, including atopic dermatitis [29, 30].

A previous study indicated that MRGPRX2-mediated mast cell activation contributed to local inflammation and sensory neuron excitation in atopic dermatitis [31]. In addition, ORAI channels contributed to MRGPRX2-mediated mast cell activation and the inactivation of mast cells might provide a novel treatment for neuropeptide substance P-induced atopic dermatitis [13]. However, these two studies lack *in vivo* experiment validation. Other publications related with the roles of MRGPRX2-mediated mast cells in atopic dermatitis are mainly reviews [10, 32]. In this study, we firstly found that Celastrol remarkably inhibited the number of mast cells and histamine release, which might be the regulatory mechanism of Celastrol to modulate scratching. However, the treatment with MRGPRX2^OE^ greatly reversed the influence of Celastrol, suggesting that Celastrol might regulate mast cell production and histamine release through targeting MRGPRX2.

It was reported that ORAI channel was involved in MRGPRX2-regulated mast cell activation, and inhibition of it might be the potential treatment method for MRGPRX2-mediated disorders [13]. Meanwhile, Tribuli Fructus extract could suppress skin inflammation in atopic dermatitis mice through regulating calcium channels and mast cell activation by targeting ORAI-1 [13, 33]. In this study, we found that the protein expression levels of MRGPRX2, ORAI-1, and ORAI-2 in the skin tissues were greatly suppressed by Celastrol, but the decrease of MRGPRX2/ORAI was promoted by MRGPRX2^OE^.

## 5. Limitations

There are several limitations in this research. Firstly, the influence of Celastrol on atopic dermatitis through the MRGPRX2/ORAI axis needs to be validated with human atopic dermatitis tissues. Secondly, further targeting signaling pathway needs to be further explored. Thirdly, the specific functioning microRNA needs to be studied.

## 6. Conclusion

In the present study, we demonstrated that Celastrol could greatly inhibit atopic dermatitis-related tissue injury, mast cell production, histamine release, scratching level, inflammatory factor expression, and activation of the MRGPRX2/ORAI axis in the DNFB-induced atopic dermatitis model. However, the regulatory role of Celastrol was remarkably reversed by overexpression of MRGPRX2, indicating that Celastrol might regulate atopic dermatitis through targeting MRGPRX2.

## Data Availability

The datasets used in the current study are available from the corresponding author on reasonable request.
